# Percutaneous endovascular management of recurrent aneurysm of transplant renal artery anastomosed to internal iliac artery

**DOI:** 10.4103/0970-1591.42629

**Published:** 2008

**Authors:** Umapati N. Hegde, Mohan M. Rajapurkar, Sishir D. Gang, Suhas S. Lele

**Affiliations:** Department of Nephrology, Muljibhai Patel Society for Research in Nephro Urology, Dr. Virendra Desai Road, Nadiad-387 001, Gujarat, India; 1 Department of Cardiology, Bhailal Amin General Hospital, Vadodara, Gujarat, India

**Keywords:** Aneurysm, covered stent-graft, endovascular management, transplant renal artery

## Abstract

Aneurysm formation constitutes 0.5 to 1% of all vascular complications in transplant patients. Aneurysms may result from infection, injury during procurement or preservation, faulty suture technique or trauma. Transplant renal artery aneurysm presents with hypertension, graft dysfunction and bleeding. We report a case of percutaneous covered stent-graft for recurrent aneurysm with stenosis of transplant renal artery. To our knowledge this is the first report of successful treatment of transplant renal artery aneurysm with covered stent-graft.

## INTRODUCTION

Aneurysm formation in the transplant renal artery is an infrequent complication comprising less than 1% of the vascular complications.[1] It commonly presents with hypertension, graft dysfunction and may have a significant risk of bleeding due to rupture. We report a case of recurrent aneurysm of the transplant renal artery, which was treated successfully with percutaneous covered stent-graft.

## CASE REPORT

A 22-year-old gentleman had undergone live renal transplantation (mother donor) 20 months ago. Graft artery was anastomosed to right external iliac artery (EIA). He had acute rejection on eighth postoperative day, which was treated with pulse steroid and discharged with serum creatinine (S.Cr) of 1.2 mg/dl. Abdominal ultrasound done for the evaluation of fever (48th postoperative day), showed aneurysm of the EIA at the site of anastomosis. He underwent aneurysmectomy with ligation of EIA. Graft artery was re-anastomosed to the right internal iliac artery and femoro-femoral bypass was done using saphenous vein graft. Patient was asymptomatic for one year (S.Cr 1.5- 1.8 mg/dl). Patient started developing edema, hypertension and his S.Cr was 2.3 mg/dl and ultrasound showed aneurysm of the transplant renal artery. He was referred to our hospital for recurrent aneurysm.

**Figure 1:**
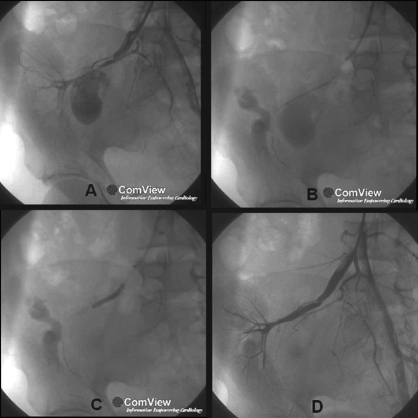
Fluoroscopic images: A- Aneurysm and stenosis of the graft artery. B- Undeployed stent-graft in place. C- Inflated balloon. D- After angioplasty and covered stent-graft placement

In our hospital, he had edema, was afebrile, hypertensive with systolo-diastolic bruit over the renal graft. His S.Cr was 3.1 mg/dl. Urine and blood culture were negative (aerobic, anaerobic, fungi). Doppler showed thick-walled aneurysm of the transplant renal artery with decreased graft perfusion. Digital subtraction angiography (DSA) revealed an aneurysm of 4 X 2.8 cm size involving transplant renal artery with 60% stenosis proximal to the aneurysm. There was only 0.5 cm of normal renal artery seen distal to the aneurysm [Figure 1A]. Surgical opinion was that surgical intervention might lead to graft nephrectomy. The graft biopsy showed no rejection (acute or chronic) and there were viable glomeruli, hence the graft dysfunction was considered to be ischemic in origin. A covered stent-graft [SYMBIOT SOE 5/45.0(OUS) - Boston Scientific International B.V], which would cover the proximal stenotic lesion and the opening of the aneurysm, was deployed under DSA [Figure 1B,C]. The technical success of the procedure was confirmed by repeat angiogram [Figure 1D].

Post procedure he was started on antiplatelet, antibiotic and antifungal prophylaxis. His edema feet decreased; blood pressure was controlled with one antihypertensive drug. S.Cr at one month and at three months was 1.9 mg/dl and 1.2 mg/dl with no blood flow in the aneurysm.

## DISCUSSION

Vascular complications account for 3-10% of all post-transplantation complications.[2] These may result in hypertension, functional impairment or loss of allograft. Aneurysm formation constitutes 0.5 to 1% of all vascular complications in transplant patients.[1] Aneurysms may result from infections, either bacterial or fungal (mycotic aneurysm), injury during procurement or preservation, ischemic damage due to striping of vasavasorum of the artery, faulty suture technique and external trauma.[1] Intimal rupture is followed by disruption of the arterial wall due to infection or trauma present in the early period of transplant, and atherosclerotic aneurysms present later in the course of transplant. In this case the exact etiology was not known. Infective etiology can't be ruled out completely even though the blood and urine culture were negative.

Aneurysm presents as recent onset or accelerated hypertension and may have deterioration of graft function. Seventy to ninety per cent of renal artery aneurysms have hypertension,[3] which may be due to: associated arterial stenosis, thromboembolism, and branch artery compression and steal syndrome.[3] Altered renal arterial hemodynamics distal to the aneurysm resulting in renin release might also cause hypertension.[4] Our patient also presented with hypertension and deterioration of graft function. There was improvement in graft function along with better blood pressure control after endovascular treatment.

Natural history of aneurysm in the transplant renal artery is not well known. In native kidney incidence of aneurismal rupture may vary from 0%[4] when it is less than 2 cm to 24%[5] when it is more than 2.5 cm. Indication for interventions are the size (> 2.5 cm), poorly calcified, thin-walled and gradually expanding aneurysms to facilitate blood pressure control, preservation of the graft and to save from the life-threatening hemorrhage due to aneurismal rupture.

Our patient had recurrent aneurysm of the transplant renal artery; first he was treated surgically. The second time the aneurysm was associated with graft dysfunction and worsening of blood pressure, without any evidence of active infection. The surgical option was considered but was deferred due to possibility of graft loss and percutaneous covered stent-graft was preferred. Covered stent-graft was successfully used percutaneously for the treatment of stenosis and aneurysm in the transplant renal artery in this patient. Post-intervention graft perfusion improved confirmed by angiography. Graft function (S.Cr) improved from 3.1 mg/dl to 1.2 mg/dl at three months.

Surgical treatment of aneurysm in post-transplant situation may lead to acute tubular necrosis, as it is associated with interruption in the blood flow to the graft. Endovascular repair greatly minimizes the ischemic time by maintaining the continuous perfusion of graft throughout the procedure except during the time of balloon expansion.

Surgery for the aneurysm may be excision of aneurysm and revascularization, autotransplantation or graft nephrectomy. Auto transplantation of the allograft into the contralateral iliac fossa may be done using cold storage or extra-corporeal continuous hypothermic perfusion for protection to preserve the renal tissue from the ischemic injury. Our patient received surgical treatment for the first time and repeat surgery for the repair of the aneurysm carried high risk of graft loss. The covered stent-graft has been used for treatment of native renal artery and aortic aneurysms and also in dissection of transplant renal artery. To our knowledge this is the first report of successful treatment of transplant renal artery aneurysm with covered stent-graft.

In conclusion, covered stent-graft placement is a possible alternative to surgery in high-risk patients to salvage the graft for the management of aneurysm of transplant renal artery.

## References

[1] Novik AC (1994). Secondary renal vascular reconstruction for arterial disease in the native and transplant kidney. Urol Clinic North Am.

[2] Campbell SC, Gill I, Novik AC (1993). Delayed allograft autotransplantation after excision of a large symptomatic renal artery pseudoaneurysm. J Urol.

[3] Youkey JR, Collins GJ, Orecchia PM, Brigham RA, Salander JM, Rich NM (1985). Saccular renal artery aneurysm as a cause of hypertension. Surgery.

[4] Mulderije ED, Berden JH, Buskens FG, Raaijmakers PA, Rosenbusch G (1985). False and true aneurysms of the renal artery after transplantation: A report of two cases. Br J Radiol.

[5] Harrow BR, Sloane JA (1959). Aneurysms of renal artery: Report of five cases. J Urol.

